# High-Resolution Identification of Specificity Determining Positions in the LacI Protein Family Using Ensembles of Sub-Sampled Alignments

**DOI:** 10.1371/journal.pone.0162579

**Published:** 2016-09-28

**Authors:** Roman Sloutsky, Kristen M. Naegle

**Affiliations:** 1 Biomedical Engineering Department, Washington University in St. Louis, St. Louis, Missouri, 63130, United States of America; 2 Center for Biological Systems Engineering, Washington University in St. Louis, St. Louis, Missouri, 63130, United States of America; University of Edinburgh, UNITED KINGDOM

## Abstract

Since the advent of large-scale genomic sequencing, and the consequent availability of large numbers of homologous protein sequences, there has been burgeoning development of methods for extracting functional information from multiple sequence alignments (MSAs). One type of analysis seeks to identify specificity determining positions (SDPs) based on the assumption that such positions are highly conserved within groups of sequences sharing functional specificity, but conserved to different amino acids in different specificity groups. This unsupervised approach to utilizing evolutionary information may elucidate mechanisms of specificity in protein-protein interactions, catalytic activity of enzymes, sensitivity to allosteric regulation, and other types of protein functionality. We present an analysis of SDPs in the LacI family of transcriptional regulators in which we 1) relax the constraint that all specificity groups must contribute to SDP signal, and 2) use a novel approach to robust treatment of sequence alignment uncertainty based on sub-sampling. We find that the vast majority of SDP signal occurs at positions with a conservation pattern that significantly complicates detection by previously described methods. This pattern, which we term “partial SDP”, consists of the commonly accepted SDP conservation pattern among a subset of specificity groups and strong degeneracy among the rest. An upshot of this fact is that the SDP complement of every specificity group appears to be unique. Additionally, sub-sampling gives us the ability to assign a confidence interval to the SDP score, as well as increase fidelity, as compared to analysis of a single, comprehensive alignment—the current standard in multiple sequence alignment methodologies.

## Introduction

Rapid advances in DNA sequencing technologies in recent decades have enabled an exponential increase in the number of fully sequenced genomes. Combined with advances in automated gene annotation and functional assignment [1–3], this has resulted in the availability of homologous protein sequences from thousands of species. This abundance of sequence data, in turn, motivated development of numerous computational strategies for inferring functional roles of individual protein residues from the amino acid composition patterns of multiple sequence alignment (MSA) columns.

One such type of analysis seeks to identify residues responsible for specificity differences in families of homologous proteins that share a common function, but differ in substrate, ligand, protein interaction partner, or various other forms of specificity. Starting with the model, first postulated by Susumu Ohno in his seminal book [4], that specificity diversification occurs through gene duplication followed by specialization of each duplicate, the approach further pre-supposes that such specificity-determining positions (SDPs) experience a specific pattern of substitutions following duplication. While positions responsible for their common function remain under constant purifying selection in both duplicates, and positions evolving neutrally diverge through random drift [5], SDPs mutate as the duplicate genes acquire new specificity, then come back under purifying selection once that specificity becomes fixed. Subsequent duplications again relax the purifying selection pressure on SDPs, followed by renewed purifying selection after further specialization. Eventually each specialized gene evolved by repeated duplication gives rise to a set of orthologs—homologs descended from speciation events—which share both the global function of the protein family and the specificity of their pre-speciation ancestor gene. In the context of SDP identification these are often called specificity groups. Positions responsible for global function remain conserved to the same amino acid across all specificity groups, while neutral positions diverge within each group. SDPs, on the other hand, remain conserved within groups due to purifying selection, but are conserved to different amino acids in each group, as required by its unique specificity. Although the numerous SDP identification algorithms [6–19] differ in their scoring functions, they all reward maximally this “conserved within specificity groups, different between” amino acid composition pattern. Because all methods agree on this, we generically refer to columns with conservation patterns approximating this ideal as having “SDP signal”.

Sub-specialization within protein families commonly involves multiple sites in a protein in a combinatorial fashion, possibly including catalytic, allosteric, and interaction sites, as well as other aspects of protein function. In a diverse protein family, each member’s specialized function is very unlikely to be determined by the same set of positions. More plausibly, positions acquire and lose specificity roles along different lineages over multiple duplications, resulting in “partial” SDPs which contribute to specialized function in some specificity groups, but not in others. Among the fraction of groups which use a particular position as an SDP, the position should exhibit a conservation pattern consistent with SDP signal. Among remaining groups purifying selection pressure will have been lost, and the position likely reverted to evolving neutrally: diverging through random drift, resulting in low conservation both within and between groups. In fact, we expect relatively few positions to be under purifying selection in all ortholog sets, with many more positions experiencing a patchwork of purifying selection and neutral evolution across different lineages. If this is the case, one expects to find many positions with a “heterogenous” conservation pattern across ortholog sets: conserved in some sets, degenerate in others. Heterogeneous conservation was previously reported by Casari et al [20] in the Ras/Rab/Rho family, in G2/M and B-type cyclins, and in a small subset of SH2 domains. In larger protein families, at least some heterogeneous positions may contain detectable SDP signal among the specificity groups in which the position is conserved—indicating that this fraction of ortholog sets use the position in a specificity-determining role. Although several methods allow limiting conservation analysis to a subset of input sequences by only considering sequences corresponding to leaves descendant from an internal node in a phylogeny [21–23], doing so assumes are relevant signal is contained in this monophyletic subset. However, a partial SDP position that acquired and lost its specificity-determining role multiple times would not have its SDP signal confined to any monophyletic subset of ortholog sets. Identifying SDPs in the context of such non-uniform evolutionary history remains a challenge to understanding specificity in large protein families.

Another, fundamental challenge to all sequence analyses requiring an input MSA, like SDP identification, comes from the uncertainty and imperfect accuracy of the alignment process itself. In all but the most trivial cases, different multiple sequence alignment tools produce differing alignments of the same collection of input sequences. And yet, subsequent downstream applications treat input alignments as an observation, assuming their correctness [24], even though a number of studies [24–32] have demonstrated sensitivity of downstream applications to alignment variability. To make matters worse, two recent studies demonstrated strong positive correlation between the number of aligned sequences and the overall amount of alignment error for every tested alignment tool [31, 33]. Furthermore, after repeatedly aligning a constant subset of sequences with different collections of additional homologs, Sievers *et al.* [33] found that the embedded alignment of the constant subset was affected by the variable additional sequences—illustrating sensitivity of pairwise alignments embedded in an MSA to the total number and context of aligned sequences. Although a number of approaches for identification and removal of alignment columns with the most uncertainty have been developed [34–38], simply removing columns is of limited utility for column-wise analyses like SDP identification. Therefore, using all available sequence data, in a manner robust to alignment uncertainty and inaccuracy, is a second challenge in SDP analysis of large protein families.

In this work we identify numerous partial SDPs in the LacI family of bacterial transcriptional regulators, previously analyzed by multiple SDP identification methods [8, 10, 12, 17, 39]. LacI family members vary in their DNA binding specificity, allosteric regulator identity and promiscuity, and even regulatory logic—with some members dissociating from DNA upon binding their regulators and others requiring their regulator to bind DNA [40]. Since the LacI family contains at least 34, possibly as many as 45 members, each represented by a set of orthologs from numerous bacterial species [41], it also poses the challenge of robustly analyzing MSAs of large collections of homologs. To address this challenge we employ sub-sampling to generate an ensemble of LacI MSAs, taking advantage of a large amount of sequence data, while aligning relatively few sequences at any one time. We extend an existing SDP identification method, *GroupSim* [19], in order to account for partial SDPs and to calculate group-specific scores—allowing us to determine whether a position is an SDP for some groups, but not for others. We find support for partial SDP in the physical interactions of corresponding side chains in solved structures of LacI and its homologs. In comparing group-specific SDP scores in our work with two other methods, SDPPred [8, 10] and Speer [17, 39], we find that group-specific scoring identifies many positions that cannot be detected by existing methods and highlights where these methods are likely making false positive SDP calls for subsets of specificity groups. Consistent with our expectation for a protein family with complex specificity, and in contrast to SDPPred, Speer, and *GroupSim*, SDP complements identified by our group-specific method vary dramatically between family members. The resulting aggregate analysis is robust to alignment uncertainty and inaccuracy, with individual sequence position results demonstrating a wide range of sensitivity to alignment variation. Our sub-sampling approach constitutes a general framework for robust treatment of any SDP method and, more generally, of any computational analysis of multiple sequence alignments.

## Results

We assembled a pool of 1814 unique sequences covering 20 members of the LacI protein family, each represented by a set of orthologs, consisting of between 28 and 192 sequences, from different bacterial species. Since a multiple sequence alignment (MSA) of this many sequences will suffer from significantly higher error [33], we opted to align a subset of 200 sequences randomly sampled from the pool. To create sufficient sampling of the full sequence space, we repeated this sub-sampling and alignment 5000 times to form an ensemble of MSAs. In order to merge analysis results across the ensemble, we included a reference sequence in each set, for a total of 201 sequences in every alignment. Results were aggregated by reference sequence position and are referenced that way throughout the text. To avoid bias the reference sequence was withheld from analysis and only the 200 sampled sequences were used. Six separate ensembles were generated, each with a respective reference sequence representing one of the six family members with a solved structure: AscG, CcpA, FruR, LacI, PurR, and TreR. Positions in reference sequences were independently mapped to each other with a structural alignment, allowing us to compare results for structurally homologous sequence positions in different family members. Because results from all six ensembles were highly similar, we report results based on the LacI reference sequence (LacI of *Escherichia coli*, UniProt accession P03023), unless otherwise specified.

Our ensemble approach allowed us to quantify the variability column-wise metrics experience as a result of differences in alignment inputs and specific errors, which will be highlighted throughout the remaining results. In short, by using the average SDP score across the ensemble, the result becomes more robust to uncertainty in the alignment process.

### Detection of SDP signal at heterogeneously conserved positions

We assume each member of the LacI family has unique specificity and, therefore, we treat sets of family member orthologs as specificity groups for the purposes of SDP analysis. This assumption is predicated on the fact that paralogs with identical function are extremely rare. Instead, when the two copies of a gene resulting from a duplication event fail to evolve functional differences, one copy tends to become a pseudogene [42].

Throughout the text “ortholog set” and “specificity group” both refer to the collection of orthologs of a family member protein from different bacterial species. “Family member” is also used to refer broadly to all orthologs of a protein.

#### Relationships between conservation, agreement, and SDP signal

**Fig 1:**
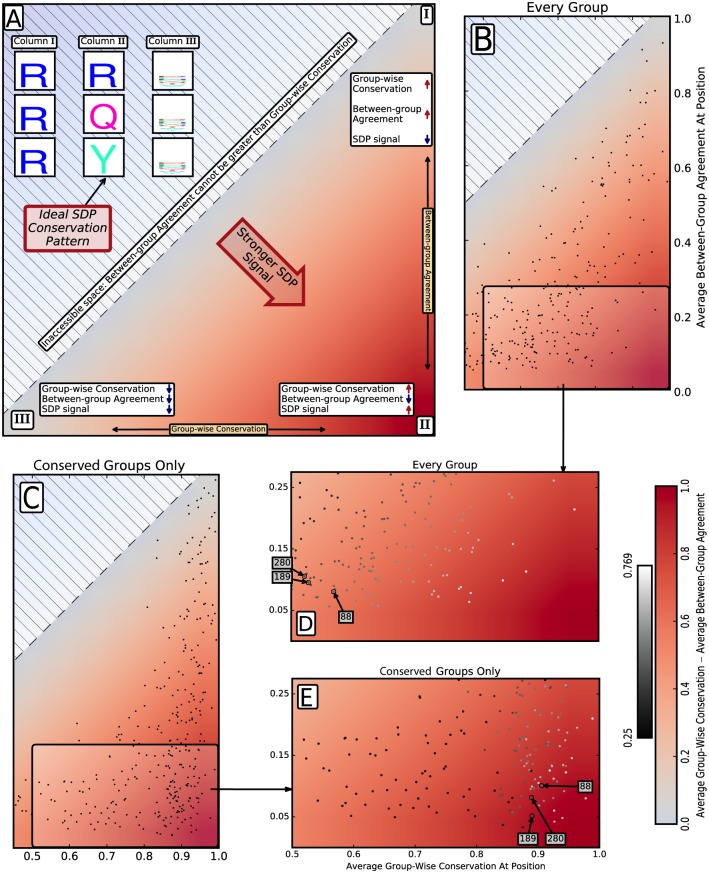
Projection into conservation-agreement space. In every panel, the color gradient represents strength of SDP signal, as quantified by average group-wise conservation minus average between-group agreement. Dark red (bottom right quadrant) represents maximal SDP signal. (A) Projections of hypothetical alignment columns for illustration: Column II has maximal SDP signal, while columns I and III have low signal. (B,C,D,E) Projections of LacI reference sequence positions with group-wise conservation and between-group agreement computed either (B,D) over every specificity group or (C,E) over conserved groups only, where group conservation is >0.6. (D) Points corresponding to LacI positions are colored in grayscale corresponding to the red color gradient of (B). (E) Points are positioned according their SDP signal calculated over conserved groups only, but using the grayscale of (D) for illustration of the shift individual sequence positions undergo as a result of the altered scoring scheme of (C).

We find it useful to represent alignment columns as points projected into a two-dimensional space—where the first dimension is the variable quantifying net amino acid conservation within specificity groups (group-wise conservation) and the second dimension is the variable quantifying net agreement between amino acid compositions of groups (between-group agreement) (Fig 1). This projection is conceptually similar to the two entropies projection, total column entropy and sum of entropies of each specificity group, used by Ye *et al.* [14]. We then calculate SDP signal according to the method in *GroupSim* [19], defined as the difference between group-wise conservation and between-group agreement.

Fig 1(A) illustrates the fundamental relationships between group-wise conservation, between-group agreement, and SDP signal in the two-dimensional space. Conservation is maximal and agreement is minimal when every specificity group is strictly conserved to a different amino acid—the ideal SDP pattern (Fig 1, Column II). Regardless of its specific scoring function, every SDP identification method awards its maximum score to alignment columns with this pattern. Similarly, every method awards a low SDP score to columns where every group is conserved to the same amino acid (Fig 1, Column I): high conservation and high agreement, since it is proposed such positions cannot determine specificity differences. Low SDP signal is also assigned when most groups are degenerate (Fig 1, Column III)—i.e. conservation is a mandatory component of SDP signal. The consequence of this requirement is that the larger the fraction of degenerate groups, the more the SDP signal degrades.

#### Quantifying group conservation and between-group agreement across the ensemble

**Fig 2:**
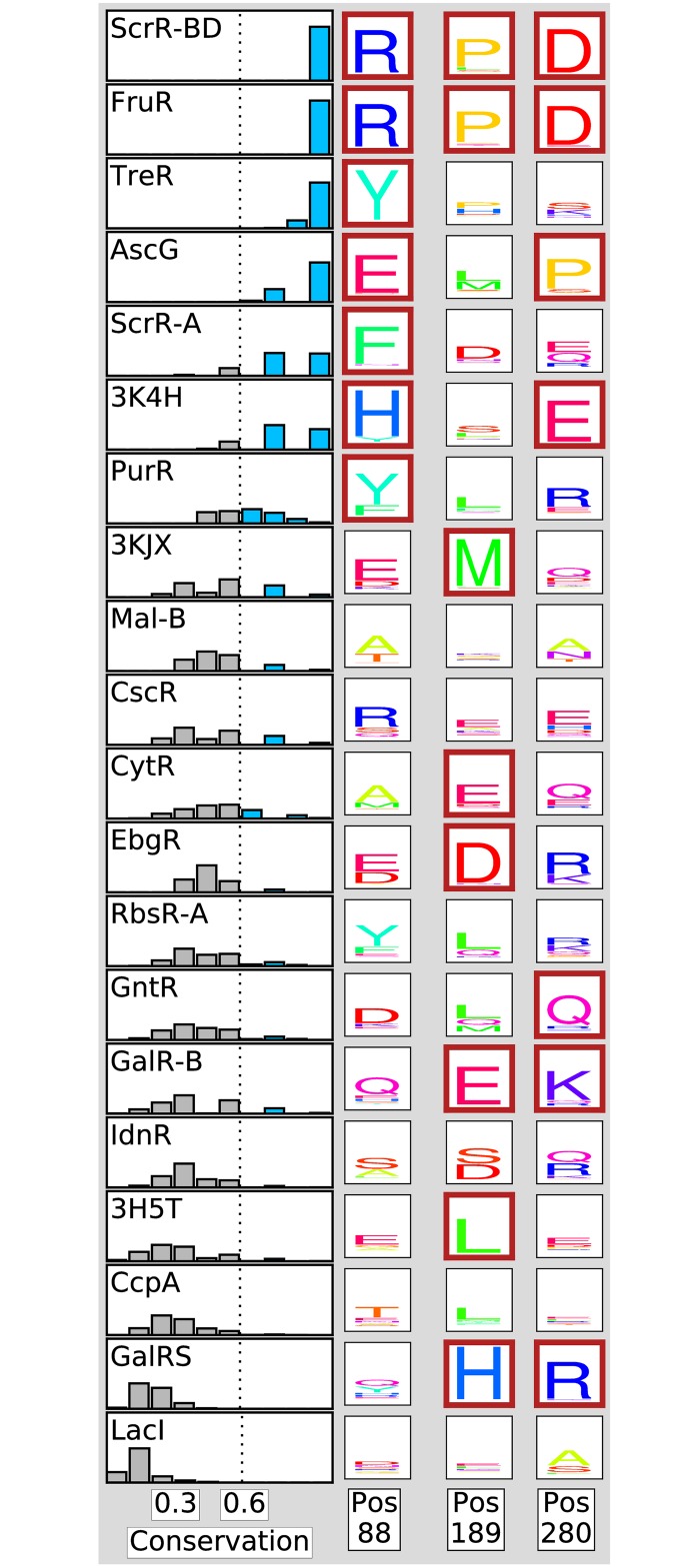
Amino acid composition at heterogeneously conserved positions. Historgrams at left show group conservation distributions at position 88 over the MSA ensemble for each family member. The dotted line indicates threshold for “conserved” designation, separating high conservation in blue from low conservation in gray. Amino acid content of each of the 20 ortholog sets is represented by sequence logos for three positions that demonstrate heterogeneous conservation. Rows correspond to LacI family members. Sequence logos for ortholog sets with average group conservation above the conservation cutoff are outlined in maroon.

Analysis of any property of an alignment column can be extended across an ensemble of alignments. A benefit of the ensemble approach is the ability to explore the distribution of a property over collections of input sequences. For example, Fig 2 demonstrates the distributions of conservation within each of the 20 ortholog sets representing 20 LacI family members for a single position (LacI reference position 88). In almost all cases there is variability in this calculation (the only exceptions are the strictly conserved scR-BD and fruR families). By taking the average value for conservation and agreement, we are, ideally, creating robustness to the variability of these metrics as a function of alignment.

In order to establish a metric for high conservation within a specificity group across the ensemble, we call a group conserved if its average conservation score is 0.6 or greater. For a group of eight sequences, this threshold corresponds to six or more amino acids being identical. In Fig 2 ScrR-BD and FruR orthologs are most conserved at reference position 88, with conservation of 1.0 in every ensemble alignment, while LacI orthologs are least conserved, and consistently so across the ensemble. We define a column as heterogeneously conserved, or heterogeneous, when specificity groups in it span conservation extremes: at least six groups have conservation greater than 0.6 and at least six others have conservation less than 0.5.

#### Conservation heterogeneity is pervasive

A third of LacI reference sequence positions (124 of 360) exhibit heterogeneous group conservation. We represent both conservation and amino acid content over the ensemble at three positions with heterogeneous conservation (positions 88, 189, and 280) by sequence logos [43, 44] (Fig 2). The subset of conserved groups varies dramatically from one heterogeneous position to another. On average, a specificity group is conserved at only 55 of 124 positions, and no group is conserved at more than 82 positions, suggesting that purifying selection pressure is acting on a unique subset of positions in each ortholog set.

#### Noise from degenerate groups masks strong SDP signal at some positions

Plotting reference sequence positions in conservation-agreement space illustrates the impact of conservation heterogeneity on SDP signal across all positions (Fig 1(B) and 1(D)). Since so much of the LacI sequence is heterogeneously conserved across family members, the area of strongest SDP signal (bottom right quadrant Fig 1(B)) is relatively unpopulated. Noise from degenerate groups hampers detection of SDP signal among conserved groups by lowering group-wise conservation and making the position as a whole indistinguishable from positions with uniformly lower conservation across all groups.

In Fig 1(C) and 1(E)) amino acid positions are re-plotted according to a calculation including only the subset of groups identified as being conserved (group conservation ≥ 0.6) at a position. This process ideally removes the noise contributed by degenerate groups normally included in traditional SDP calculations. Naturally, when only conserved groups are considered, group-wise conservation increases for all positions, except those at which every group is conserved—resulting in a shift of all positions to the right. However, comparing Fig 1(D) and 1(E) demonstrates that this shift is far from homogeneous. Two color gradients are used in order to compare the original, all group calculation, with the calculation based only on the subset of groups that demonstrate conservation. In Fig 1(E), where positions are plotted by conserved groups only, the area of strongest SDP signal is populated by a mixture of points having variable SDP signal in the original scoring scheme. For example, positions 88, 189, and 280, whose group amino acid composition is shown in Fig 2, are three of the biggest beneficiaries of the modified scoring scheme. While removing noise from degenerate groups increases SDP signal overall, individual positions still vary in the strength of signal among their conserved groups. Based on this analysis, we incorporated this filter into a high-resolution SDP metric.

### Detection of SDP signal in individual specificity groups

As expected for a diverse protein family, the vast majority of noise-filtered SDP signal in the LacI family is contributed by positions with high heterogeneity of conservation, i.e. positions at which a subset of specificity groups are degenerate and another subset of groups are conserved. We propose a simple method for identifying partial SDPs by evaluating SDP signal in a group-specific manner. Here, we compare the results of this approach to three existing methods, SDPPred, Speer, and *GroupSim*, which—like all existing methods—assign a single score to every specificity group in an alignment column. Our results suggest that the standard approach can produce both false positives and false negatives as a result of heterogeneous conservation across groups.

#### A group-specific SDP score

We compute a modified *GroupSim* score, filtered for noise from degenerate groups by only including conserved groups, where group conservation ≥ 0.6, in the score calculation. We refer to these conserved groups as “support” groups, since only these groups can provide support for an SDP call. For each specificity group in an alignment column, we then modulate the score by a weight that accounts for the evidence of the position’s importance to this group, based on the group’s conservation. Specifically this is calculated according to the following:

$$\begin{array}{cc} W_{group}\;\times & (\text{group-wise conservation over support }-\\ & \text{between-group agreement over support}) \end{array}$$ (1)

where

$$W_{group}=\left\{\begin{array}{cc} 1 & \text{if}\;\text{group}\in \text{support}\\ \text{group}\;\text{conservation} & \text{otherwise} \end{array}\right.$$ (2)

**Fig 3:**
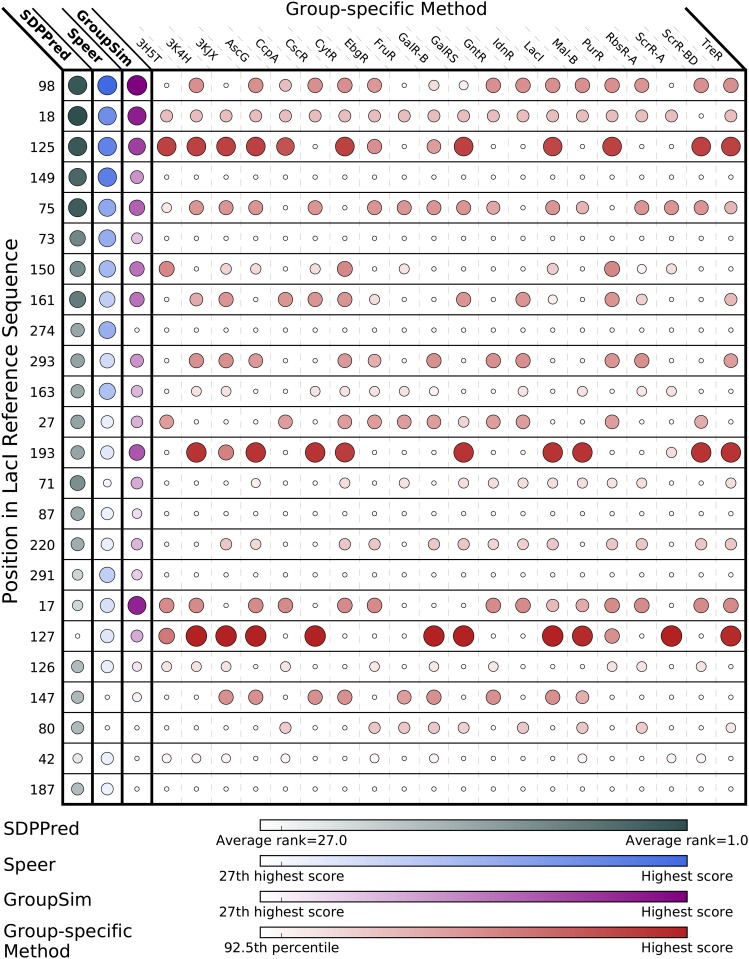
SDP results for the highest scoring positions by SDPPred and Speer. Each position receiving a top-20 score from at least one of the comparative methods, SDPPred and Speer, are shown. Ensemble score for SDPPred is the average ranking. Ensemble score for Speer is the average z-score. See Methods for details on SDPPred and Speer ensemble averages. Position scores are shown for SDPPred, Speer, and *GroupSim*. Group-specific scores for each specificity group at the corresponding position are also shown. Marker size and color correspond to score according to color bars. Note that top 7.5% of scores make up the vast majority of color scale for each method. For column-wise scoring methods the 27th highest score corresponds to the 92.5th percentile, since 27 ÷ 360 = 0.075, or 7.5%.

**Fig 4:**
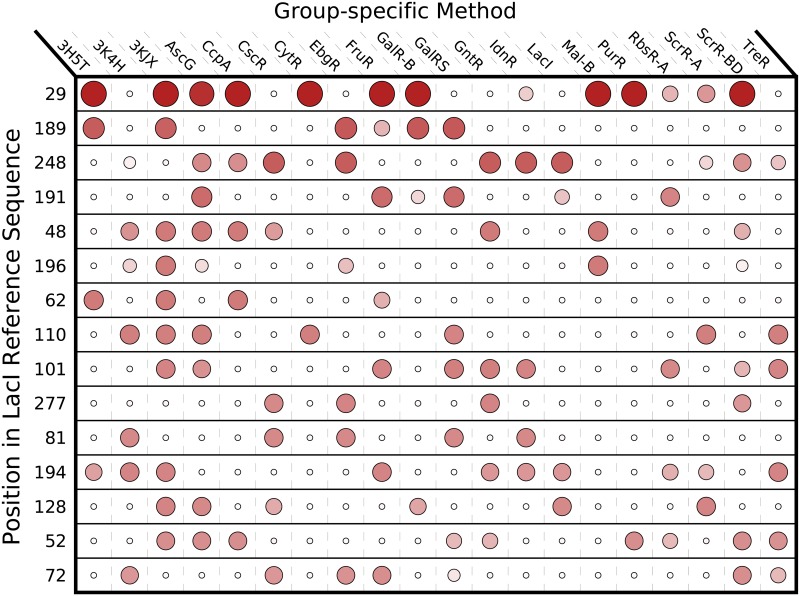
Group-specific SDP signal undetected by SDPPred or Speer. Marker size and color corresponds to group-specific score according to color bar in Fig 3.

Averaging this score over the ensemble of MSAs accounts for heterogeneity in a group’s conservation. Groups conserved at a position in every ensemble MSA receive a higher score than groups conserved at the position in a fraction of MSAs. The outcome of this approach is an individualized score for every specificity group (Figs 3 and 4).

#### SDP signal is highly variable across specificity groups

We compare results of our group-specific method with the *GroupSim* method, on which our method is based, and with two other existing methods, SDPPred and Speer, for the 20 highest scoring LacI sequence positions as judged by either of the latter two methods (Fig 3). SDPPred, Speer, and *GroupSim* scores for a position apply to every specificity group. Overlap between SDPPred, Speer, and *GroupSim* is high—16 positions are among the top 20 for all three methods—confirming that different methods generally detect the same SDP signal. However, group-specific scoring demonstrates that SDP signal, defined as being in the top 7.5% of all group-specific scores, is never uniformly high across all specificity groups. SDP signal detected by SDPPred, Speer, or *GroupSim* is supported by, on average, only 12 of 20 specificity groups. Therefore, the group-specific scoring scheme is able to identify groups with low SDP signal due to low conservation. Given that conservation within a specificity group is a requisite for hypothetical importance in a specificity determining role, it is likely that traditional methods are overcalling SDPs at these positions for those groups and a group-specific scoring scheme rectifies this.

Our method identified 15 additional LacI positions with strong SDP signal, where at least one group’s score is in the top 5% of all group-specific scores (Fig 4). All of these positions score outside the top 20 for both SDPPred and Speer, likely due to the fact that, on average, only 7.4 of 20 groups have detectable signal in this set. Position 29 scores 11th highest with *GroupSim*, underscoring the modest differences between existing methods, but the remaining 14 positions in Fig 4 score outside of the top 20 for *GroupSim* as well. Noise from numerous degenerate groups masks the SDP signal at these positions when SDP is calculated as a property of all groups. Our group-specific method detects partial SDPs even when the signal is present in a small fraction of specificity groups.

Figs 3 and 4 offer a striking illustration of the complexity of specificity encoding in LacI family proteins. Every single position with detectable signal is a partial SDP to some extent, and no two positions appear to have signal in the same subset of family members. There are some positions (62, 81, 128, 189, 191, 196, and 277) that additionally highlight the sensitivity of SDP analysis to available sequence data, since all of these positions would have failed to have high SDP signal, should the latter three ortholog sets not been included in this analysis. Non-inclusion of a group could easily occur if there was low representation of these orthologs in currently sequenced species. This highlights the sensitivity of SDP analysis to input and importance of using all available data.

A subset of positions score among the top 20 with either SDPPred or Speer, but outside of the top 7.5% for our group-specific method: 149, 73, 274, 87, 291, and 187. Of these, 149, 73, 87, and 291, but not 274 or 187 score in the top 7.5% for *GroupSim* (Fig 3), though *GroupSim* scores each position lower than SDPPred or Speer. The fact that *GroupSim* ranks these positions higher than the group-specific method is misleading: group-specific scores for conserved groups at these positions are actually *higher* than *GroupSim* scores (because the two methods use the same scoring function, scores can be compared directly). However, because their SDP signal is selectively boosted by the noise filtering in our method, positions in Fig 4 crowd positions 149, 73, 87, and 291 outside of the top 7.5%. The group-specific method prioritizes positions that are very different from those prioritized by existing methods.

From exploring the similarities among positions ranked higher by other methods than by our group-specific method (S1 Fig), a clear pattern emerges: strength of SDP signal detected by any method falls as the fraction of groups conserved to the same amino acid increases, resulting in greater between-group agreement. While SDPPred, Speer, and *GroupSim* detect SDP signal at some or all of these positions, none of the four methods detect signal at positions 22 or 25 (S1 Fig). In addition, it appears that the *GroupSim* scoring function penalizes between-group agreement somewhat more severely than those of SDPPred and Speer, explaining why each position with this pattern is ranked lower by *GroupSim* (Fig 3). Detecting SDP signal in conservation patterns like positions 22 and 25, at which a large fraction of groups are conserved to the same amino acid, presents a considerable challenge to all SDP identification methods.

### Structural organization of group-specific SDPs

**Fig 5:**
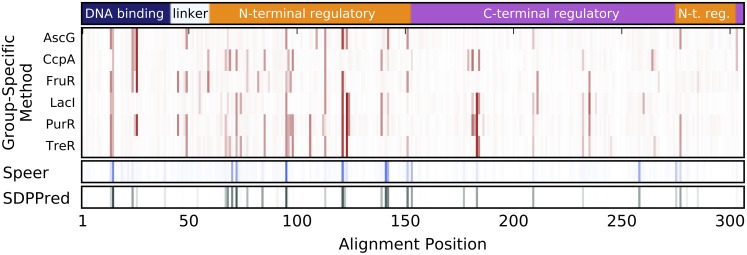
SDP scores mapped onto reference structural alignment. Locations of alignment positions in structural features are indicated in the top track. The allosteric site is located at the interface of N-terminal and C-terminal regulatory sub-domains, each of which is split into two linear segments of the polypeptide chain, as indicated. Heatmap colors correspond to group-specific scores for indicated specificity groups and whole-position Speer and SDPPred scores, according to the color bars in Fig 3.

**Fig 6:**
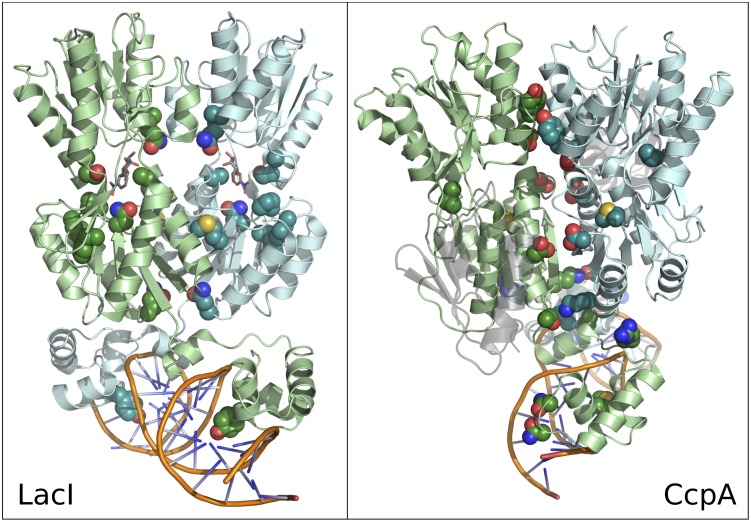
Structural distribution of SDP complements of LacI and CcpA. LacI (left) and CcpA (right) SDPs scoring in top 5% of all group-specific SDP highlighted on LacI (2pe5) and CcpA (3oqo) structures. Each protein is shown as a homo-dimer complexed with DNA, with one monomer shown in blue and the other in green. SDP side chains shown in space-filling representation in color matching their monomer. LacI ligand and CcpA binding partner protein shown in gray. CcpA binding partner is semi-transparent.

The position of a residue in the 3-dimensional structure of a protein can provide clues to its role in protein function and specificity. Therefore, we explored SDP positions for those families where structures are available. We mapped positions scoring in the top 5% of all group-specific SDP scores onto family members with solved structures (Figs 5 and 6, S2, S3, S4 and S5 Figs). Based on group-specific scores, this results in a unique structural collection of SDPs for each family member.

#### SDP complements of family members have unique structural organization

In order to compare SDPs in their sequence alignments to structure, we created a structural sequence alignment of the AscG, CcpA, FruR, LacI, PurR, and TreR reference sequences and compared this to SDP scores (Fig 5). There is, overall, substantially more SDP signal in the N-terminal half of the alignment, corresponding to the helix-turn-helix DNA binding subdomain, the inter-domain linker, and the N-terminal regulatory subdomain. Together these account for DNA binding functionality and, most likely, the conformational transition induced by binding and dissociation of the allosteric regulator. In addition, several SDPs in the C-terminal regulatory subdomain are in the allosteric site located at the interface of N-terminal and C-terminal regulatory subdomains. By comparison, the remainder of the C-terminal sub-domain is relatively devoid of SDP signal.

In order to locate the positions of top-scoring SDPs within the 3-dimensional structure, we mapped SDPs onto the structures of two family members, LacI and CcpA (Fig 6). SDP complements of LacI and CcpA identified by the group-specific method clearly have different spacial organization. LacI SDPs cluster near the allosteric binding site and in the adjacent protein core region, where they are likely participate in ligand-induced conformational changes. Only a single DNA contacting residue has strong SDP signal in LacI, although additional DNA contacting residues have an SDP-like conservation pattern, impossible to detect by any of the four methods due to high between-group agreement (as discussed earlier). On the other hand, CcpA SDPs cluster almost exclusively at monomer-monomer and protein-DNA interfaces, with three SDPs contacting DNA. The prevalence of positions at the interface between monomers suggests CcpA diverged from the rest of the LacI family in some functional aspect of dimerization.

Structural maps of AscG, FruR, PurR, and TreR SDP complements are shown in S2 through S5 Figs. The comprehensive mapping of SDP signal onto available structures suggests that family members diverged through specialization in varying aspects of function, as indicated by clustering of SDPs at different locations in the protein.

#### Structural evidence that an SDP is used by only a fraction of family members

**Fig 7:**
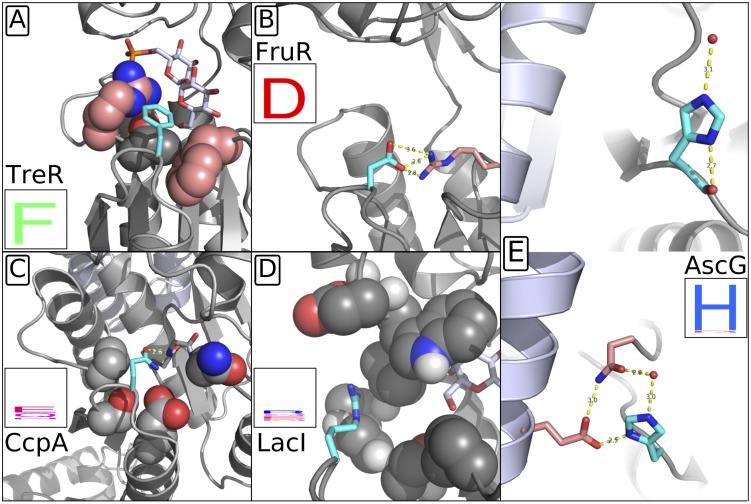
Structural evidence of partial SDP at LacI position 101. Interactions of TreR (A), FruR (B), CcpA (C), LacI (D), and AscG (E) positions corresponding to LacI position 101, according to the structural alignment. The side chain at the position homologous to LacI 101 is shown in light blue. Side chains at neighboring positions are shown in salmon, if those positions are SDPs, and in gray otherwise. Amino acid composition of the ortholog set is represented by sequence logo. Packing interaction of TreR F102 with F127 and hydrogen bonding interaction of FruR D101 with R149 are highly specific. CcpA Q101 and LacI R101 do not form specific interactions, although CcpA Q101 does participate in a single hydrogen bond. Glutamic acid and asparagine, capable of making the same interaction, also occur among CcpA orthologs. LacI R101 is exposed to solvent, and several other polar amino acids occur at the position. AscG H101 participates in two different interactions. (E), top: hydrogen bonding with cis-monomer backbone (gray) and coordinated water molecule (red dot). (E), bottom: hydrogen bond network with cis-monomeric N68, trans-monomeric E88 (light violet backbone), and another coordinated water.

In comparison to other methods, our method has increased the total number of positions with significant SDP signal. Additionally, our group-specific scoring scheme uses group conservation to identify subsets of specificity groups that are most and least likely to use the position as a specificity determinant. We illustrate our method’s ability to identify these subsets by highlighting the structural roles of residues at a position where our method identified a partial SDP—position 101 in the LacI reference sequence (Fig 7). These residues (TreR F102, FruR D101, CcpA Q101, LacI R101, and AscG H101) are homologous to each other, according to the structural alignment, and correspond to position 101 of the LacI reference sequence in our analysis. In TreR, FruR, and AscG this position is conserved to three unique amino acids and accordingly, all three received very high group-specific SDP scores. In their respective structures all three participate in highly specific hydrophobic packing (TreR) or hydrogen bonding (FruR, AscG) interactions which cannot be satisfied by other amino acids. In contrast, in LacI and CcpA this position is degenerate and receives low group-specific scores. Accordingly, R101 of LacI has no obvious interactions with either the nearby ligand or any neighboring residues, none of which are SDPs. Since the position is exposed to solvent, theoretically any polar residue should be tolerated. This is borne out by the range of amino acids occurring at this position in LacI orthologs. In CcpA Q101 forms a single hydrogen bond with a nearby backbone nitrogen atom. Again, none of the neighboring positions are SDPs. Asparagine and glutamic acid, both capable of forming the same hydrogen bond, are present at this position in other CcpA orthologs. AscG H101 presents a particularly interesting case study for this partial SDP position. Histidine is strictly conserved in AscG orthologs and the group-specific SDP score is high. In Fig 7(E), the two H101 residues in an AscG dimer participate in two different interactions—one trans and one cis—neither of which alone appears to strictly require histidine. However, only histidine can satisfy both interactions simultaneously, consistent with its conservation among AscG orthologs.

These structural observations support the hypothesis that position 101 contributes to specificities of TreR, FruR, and AscG, but not of LacI or CcpA. For AscG, although neither H101 interaction alone provides evidence supporting SDP, the two taken together are consistent with the SDP call. This example demonstrates the usefulness in group-specific scoring, which detected both the importance of position 101 to specificity groups in which it is conserved and its lack of a specific role in specificity groups in which it is degenerate.

### Sensitivity of ensemble SDP scores to alignment uncertainty

**Fig 8:**
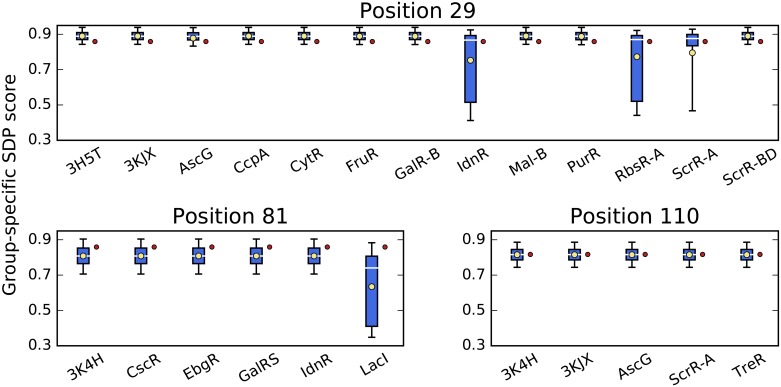
SDP score distributions vs comprehensive alignment scores. Score distributions and the comprehensive alignment scores for specificity groups with a score falling in the top 1% are plotted for positions 29, 81, and 110. Score distributions shown as box plots, with medians indicated by white lines and means indicated by yellow dots. Boxes cover middle two quartiles of score distributions, while whiskers cover middle 95%. Comprehensive alignment scores shown as red dots. These can fall below (position 29), above (position 81), or within (position 110) the middle two quartiles of the ensemble distributions. Some ortholog sets (IdnR, RbsR-A, ScrR-A at position 29, LacI at position 81) can be substantially more sensitive to alignment variability than other ortholog sets at the same position. This fact is reflected in their ensemble score (distribution average—yellow dot), but not in the comprehensive alignment score.

Results reported so far were obtained from an ensemble of MSAs. In order to compare ensemble results to the traditional single-MSA approach, we created a single, “comprehensive” alignment of all 1814 sequences and scored it with our group-specific SDP method. Even for SDP signal in the top 1%, when groups are most conserved, comprehensive alignment scores are often outliers with respect to score distributions over the ensemble (Fig 8). Consistent agreement between the average score from the ensemble and the score from the comprehensive alignment, such as seen at position 110, is rare. More often the comprehensive alignment score falls in the tails of ensemble score distributions, such as seen at positions 29 and 81.

In most cases ensemble score distributions are symmetric, as indicated by similar mean and median values of the distribution. Symmetric score distributions with low variance suggest that the same amino acid nearly always aligned to this reference sequence position for all orthologs in that specificity group. The ensemble method identifies specificity groups for which conservation varied dramatically between alignments, indicating greater uncertainty in the alignment of those orthologs at that position—e.g. IdnR and RbsR-A at position 29—and penalizes the specificity group for this uncertainty with a lower ensemble score (ensemble distribution average). The comprehensive alignment approach cannot account for different degrees of alignment uncertainty between specificity groups: all groups receive a single score.

## Discussion

In this work we demonstrated that a substantial fraction of positions in the LacI family are heterogeneously conserved—i.e. only a fraction of family members are highly conserved, while a comparable fraction are highly degenerate. In order to accurately identify the specificity determinants among positions with this conservation pattern, we implemented a scoring approach in which we 1) boost SDP signal-to-noise ratio by considering only the specificity groups that are conserved at a position and 2) modulate the score in a group-specific manner—based on each group’s degree of conservation. The paralog-specific collections of specificity determining residues identified using our method cluster on their representative protein structures in configurations that are consistent with our understanding of the functional specialization of those proteins. Importantly, the modulation of the score appears consistent with the importance of the corresponding residue, given its physical interactions. Our scoring method avoids spurious SDP identification for family members in which a position is degenerate and detects “hidden” SDPs used by a small fraction of family members.

In the course of our investigation, we encountered a conservation pattern that occurred at positions ranked significantly lower by our method than by SDPPred, Speer, or even *GroupSim*, which uses the same scoring function as our method. The pattern is characterized by conservation of a large fraction of specificity groups to the same amino acid, consistent with specialization of the common ancestor of those groups, followed by maintenance of the same functional role through the more recent duplications that gave rise to present day specificity groups. For example, at position 22, 15 of the 20 groups are conserved to arginine, while the remaining groups are conserved to one of four other amino acids. While SDPPred and Speer do tolerate a marginally greater amount of between-group agreement than the *GroupSim* scoring function, their, and *GroupSim*’s ability to rank these positions higher than our method is a side-effect of their failure to detect SDP signal at a number of positions identified by our method (Fig 3), rather than a strength. In addition, they too fail to identify positions with conservation patterns like that of positions 22 and 25 (S1 Fig) as SDPs.

Several SDP methods can simultaneously identify SDPs and optimal specificity groups [9, 12, 16, 22] by grouping sequences so that total SDP signal across all alignment columns is maximized. However, as S1 Fig illustrates, such columns often have mutually exclusive optimal sequence groupings, which further conflict with many partial SDPs identified in this work. These observations suggest that further development of SDP identification methods may be required to identify SDPs with high between-group agreement.

In this work we also tackled the common challenge of MSA-based computational analyses that arises from uncertainty of the alignment process due to both sensitivity to the input collection of sequences and to alignment error. This concern is particularly acute when analyzing large collections of sequences, because overall alignment error increases rapidly with the number of aligned sequences. We avoided making large alignments, while still taking advantage of all available sequence data, by building and analyzing ensembles of sub-sampled MSAs. Using an ensemble average improves the robustness of any metric computed on a sequence alignment and allows for the detection of regions in the alignment that may be especially prone to error. We believe this robust approach can be generalized to any analysis that requires an MSA input.

Whether “specificity determining position” is a biologically meaningful designation remains an open question. Highly targeted experiments are necessary to demonstrate this functional role: for example, by demonstrating that substituting the amino acids at these positions with the amino acids present at the homologous positions in a paralog is sufficient to switch the functional specialization of the protein to that of the paralog. The partial SDPs identified in this work, together with the ortholog sets in which these positions are conserved, will significantly reduce the number of candidates for mutation that must be considered by experimentalists when investigating specialization in the LacI family.

## Methods

### Generation of MSA ensembles

We downloaded all protein sequences from the LacI family resource AlloRep [41] and supplemented each ortholog set with sequences from EnsemblBacteria release 26 [45]. A supplemental sequence was added to an ortholog set, if: 1) it had 35% or greater identity to each ortholog in the set and 2) its lowest identity to any ortholog in the set was higher than its identity to any other sequence in the pool. We then dropped from our analysis any ortholog set containing fewer than 20 sequences in order to ensure adequate statistical coverage. The final sequence pool contains 1814 sequences split among 20 ortholog sets ranging from 28 ortholog sequences (IdnR) to 192 ortholog sequences (CcpA).

The subsamples of 200 sequences were sampled from each ortholog set according to its frequency in the full sequence set. We required a minimum allocation of eight sequences to avoid small number effects and limited the maximum to 13 sequences per ortholog set. This sampling procedure was repeated 5000 times. Each 200 sequence sample was combined with a reference sequence and the 201 sequences were aligned using MAFFT’s L-INS-i (most accurate) protocol [46, 47]. In addition to the LacI reference sequence, AscG (P24242), FruR (W8ZE48), PurR (X7PN48), and TreR (P36673) of *Escherichia coli* and CcpA (P25144) of *Bacillus subtilis* were used as reference sequences.

### SDP scoring

Pairwise comparisons between sequence positions, *comp*(*s*_1_, *s*_2_), were made using the identity matrix which had previously produced the most accurate results with both XDet [15] and *GroupSim* [19] SDP identification methods. Conservation within a specificity group was defined as the average of pairwise comparisons between all sequences in the group:

$${\left\langle \;comp(s_{1},s_{2})\;\right\rangle }_{\left\{\left(s_{1},s_{2}\right)\;\forall \;s_{1}\in group,\;\forall \;s_{2}\in group\;|\;s_{1}\neq s_{2}\right\}}$$ (3)

For an alignment column, group-wise conservation was defined as the average of each group’s conservation:

$${\left\langle \;{\left\langle \;comp(s_{1},s_{2})\;\right\rangle }_{\left\{\left(s_{1},s_{2}\right)\;\forall \;s_{1}\in group,\;\forall \;s_{2}\in group\;|\;s_{1}\neq s_{2}\right\}}\;\right\rangle }_{groups}$$ (4)

and between-group agreement was defined as the average pairwise sequence comparison between sequences belonging to different groups, averaged over all pairs of groups:

$${\left\langle \;{\left\langle \;comp(s_{1},s_{2})\;\right\rangle }_{\left\{\left(s_{1},s_{2}\right)\;\forall \;s_{1}\in g_{1},\;\forall \;s_{2}\in g_{2}\right\}}\;\right\rangle }_{\left\{\left(g_{1},g_{2}\right)\;\forall \;g_{1}\in groups,\;\forall \;g_{2}\in groups\;|\;g_{1}\neq g_{2}\right\}}$$ (5)

5000 alignments from the LacI ensemble were scored with SDPPred [8, 10], accessed via its web interface at http://bioinf.fbb.msu.ru/SDPpred/, and Speer [17, 39], downloaded from ftp://ftp.ncbi.nih.gov/pub/SPEER/ and run locally.

SDPPred produces a ranking of positions with statistically significant scores for every alignment. The number of ranked positions varies from alignment to alignment, and there is no clear way to rank positions without statistically significant scores. For each position in the LacI reference sequence we averaged its rank across all ensemble MSAs to generate an ensemble score. All positions not ranked by SDPPred for a particular MSA received the next rank after the last explicitly ranked position: e.g., if SDPPred ranked 20 positions, every unranked position received rank 21 for averaging purposes. Because of this, ensemble scores for SDPPred are not discriminatory beyond, roughly, rank 30.

Speer produces several scores, including a z-score based on the mean and variance of scores for each position in an alignment. We averaged the z-scores of each LacI position over the MSA ensemble to produce an ensemble Speer score.

### Structural mapping of SDPs

We aligned representative protein structures for each reference sequence with MUSTANG [48] to produce an independent structural alignment of the reference sequences. Structures 3dbi (AscG), 3oqo (CcpA), 2iks (FruR), 1jwl, 1tlf, 2pe5 (LacI), 1jft, 2pua (PurR), and 4xxh (TreR) were aligned. Structures with multiple ligands were used for LacI and PurR. The DNA binding subdomain and inter-domain linker segments were not included in any structures of AscG, FruR, or TreR. In order to obtain a complete mapping, full reference sequences were aligned to the structural alignment using MAFFT’s seeded alignment option.

### Implementation

Group-specific scoring code is available at http://naegle.wustl.edu/software.

## Supporting Information

S1 Fig Amino acid content at SDPs with excess between-group agreement. Amino acid content of each of 20 ortholog sets, represented by sequence logos, at positions with an SDP-like group-wise conservation pattern. Between-group agreement increases from left to right. Position 18 receives high scores from SDPPred, Speer, and the group-specific scoring method. Positions 149 through 187 are detected, with progressively lower scores, by at least one of SDPPred and Speer, but not by the group-specific method. Positions 25 and 22 are not detected by any method.
(PDF)

S2 Fig SDP complement of AscG. SDPs mapped onto structure 3brq and highlighted in space-filling representation. Structure only contains N- and C-terminal regulatory subdomains.
(PNG)

S3 Fig SDP complement of FruR. SDPs mapped onto structure 2iks and highlighted in space-filling representation. Structure only contains N- and C-terminal regulatory subdomains.
(PNG)

S4 Fig SDP complement of PurR. SDPs mapped onto structure 2puc and highlighted in space-filling representation.
(PNG)

S5 Fig SDP complement of TreR. SDPs mapped onto structure 4xxh and highlighted in space-filling representation. Structure only contains N- and C-terminal regulatory subdomains.
(PNG)
