# Supplementary material for: High-Resolution Identification of Specificity Determining Positions in the LacI Protein Family Using Ensembles of Sub-Sampled Alignments
Source: PLoS One. 2016 Sep 28;11(9):e0162579. doi: 10.1371/journal.pone.0162579 (PMC5040260; doi:10.1371/journal.pone.0162579)
Supplement: S1 Fig — Amino acid content of each of 20 ortholog sets, represented by sequence logos, at positions with an SDP-like group-wise conservation pattern. Between-group agreement increases from left to right. Position 18 receives high scores from SDPPred, Speer, and the group-specific scoring method. Positions 149 through 187 are detected, with progressively lower scores, by at least one of SDPPred and Speer, but not by the group-specific method. Positions 25 and 22 are not detected by any method. (PDF) [file pone.0162579.s001.pdf]

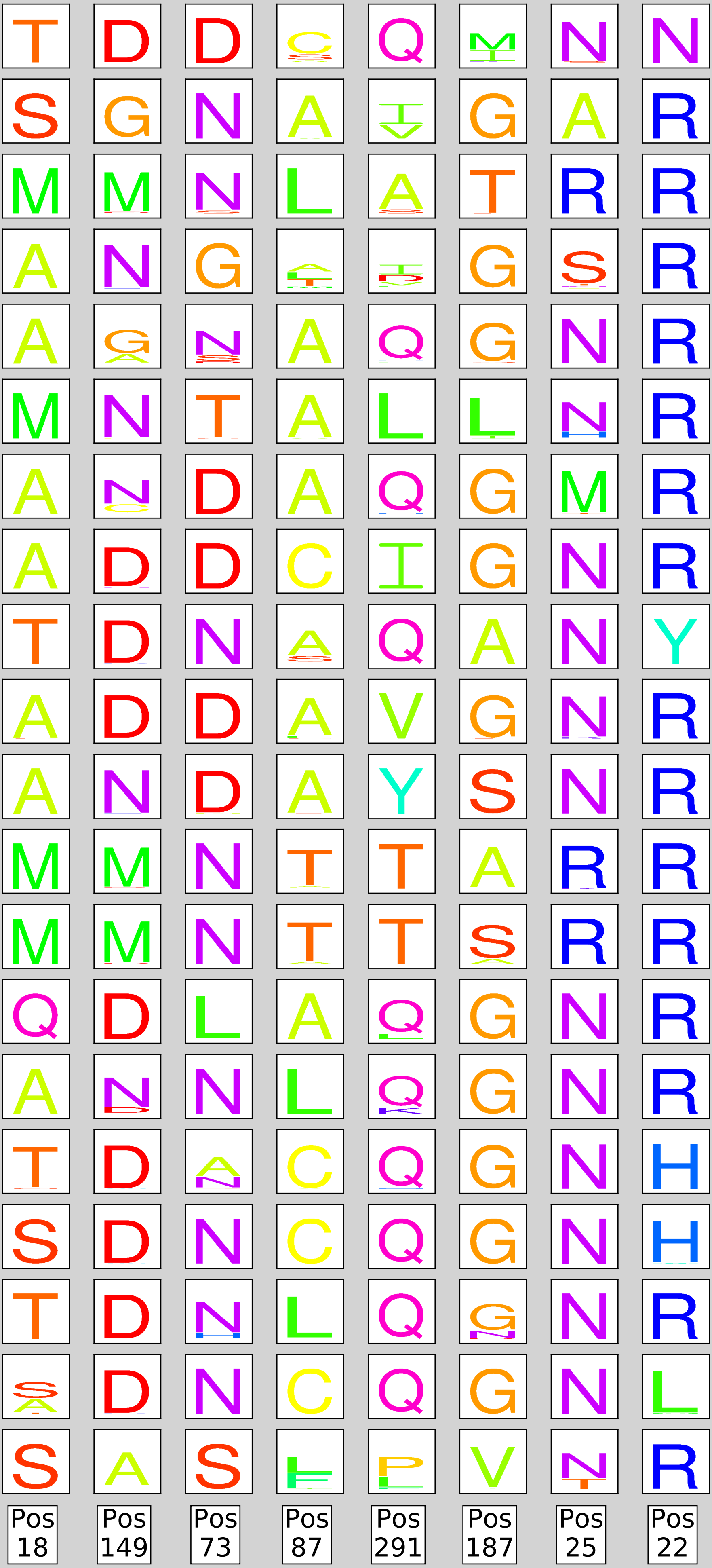
Pos 18
Pos 149
Pos 73
Pos 87
Pos 291
Pos 187
Pos 25
Pos 22
