# Supplementary figures and images for: High-Resolution Identification of Specificity Determining Positions in the LacI Protein Family Using Ensembles of Sub-Sampled Alignments
Source: PLoS One. 2016 Sep 28;11(9):e0162579. doi: 10.1371/journal.pone.0162579 (PMC5040260; doi:10.1371/journal.pone.0162579)

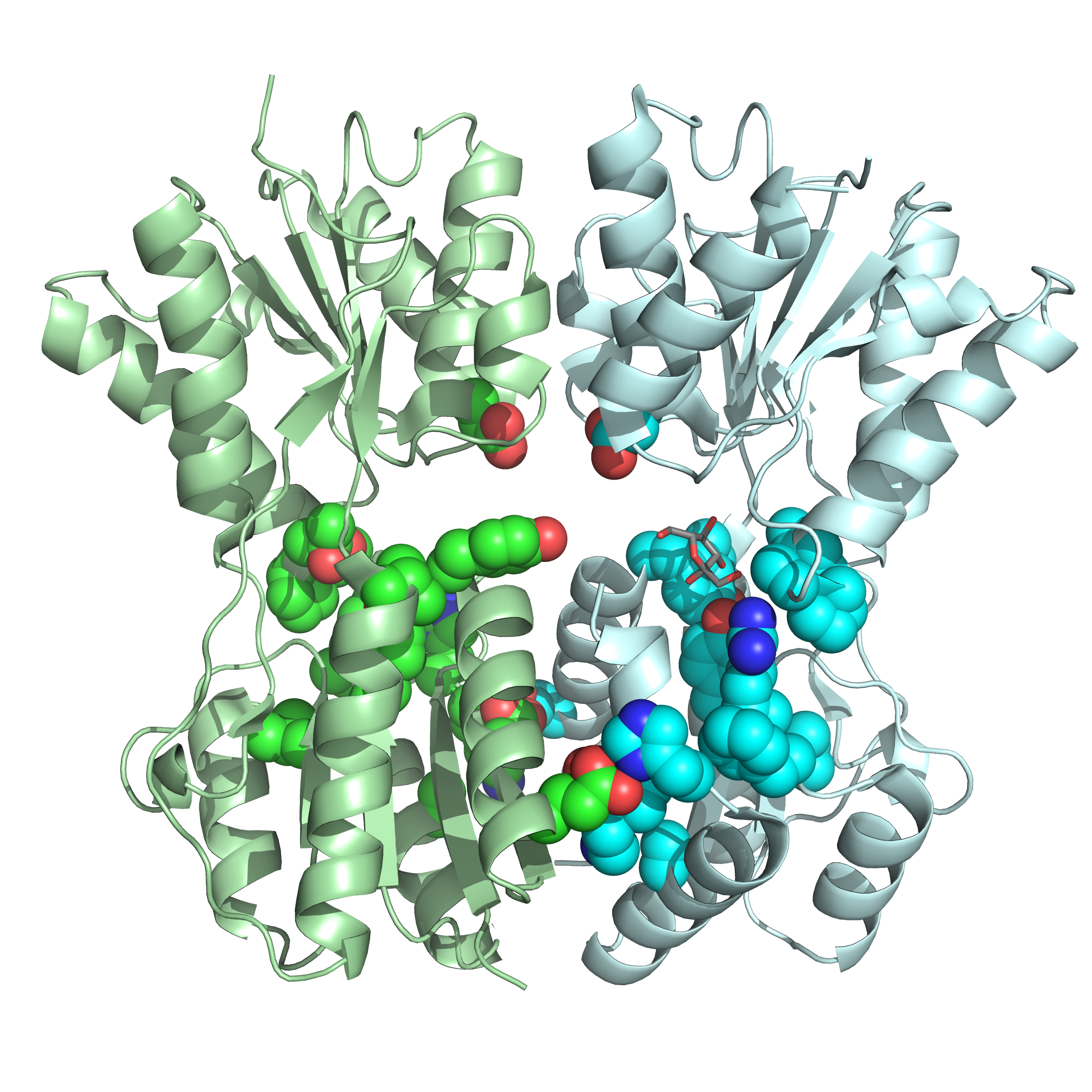

Supplement: S2 Fig — SDPs mapped onto structure 3brq and highlighted in space-filling representation. Structure only contains N- and C-terminal regulatory subdomains. (PNG) [file pone.0162579.s002.png]

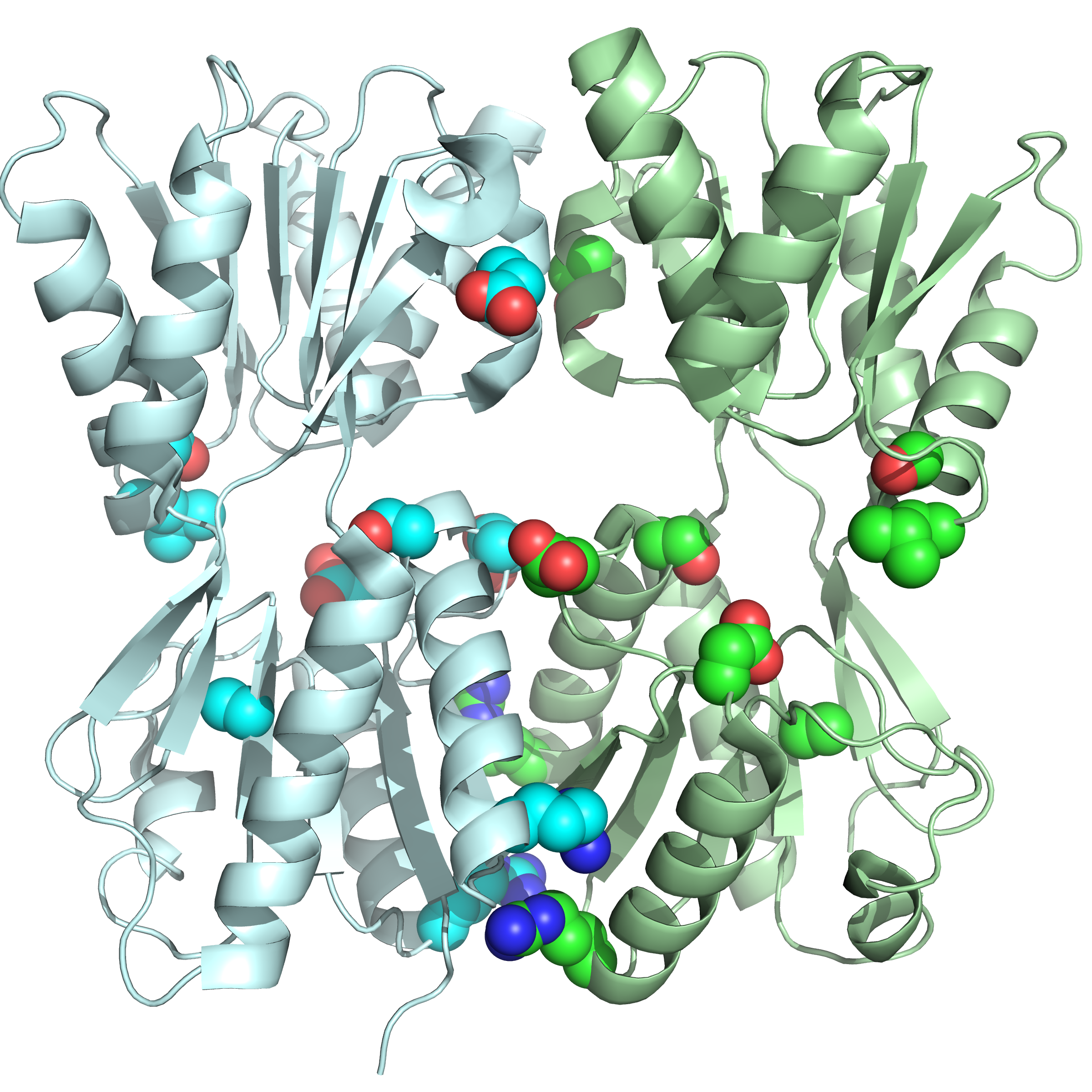

Supplement: S3 Fig — SDPs mapped onto structure 2iks and highlighted in space-filling representation. Structure only contains N- and C-terminal regulatory subdomains. (PNG) [file pone.0162579.s003.png]

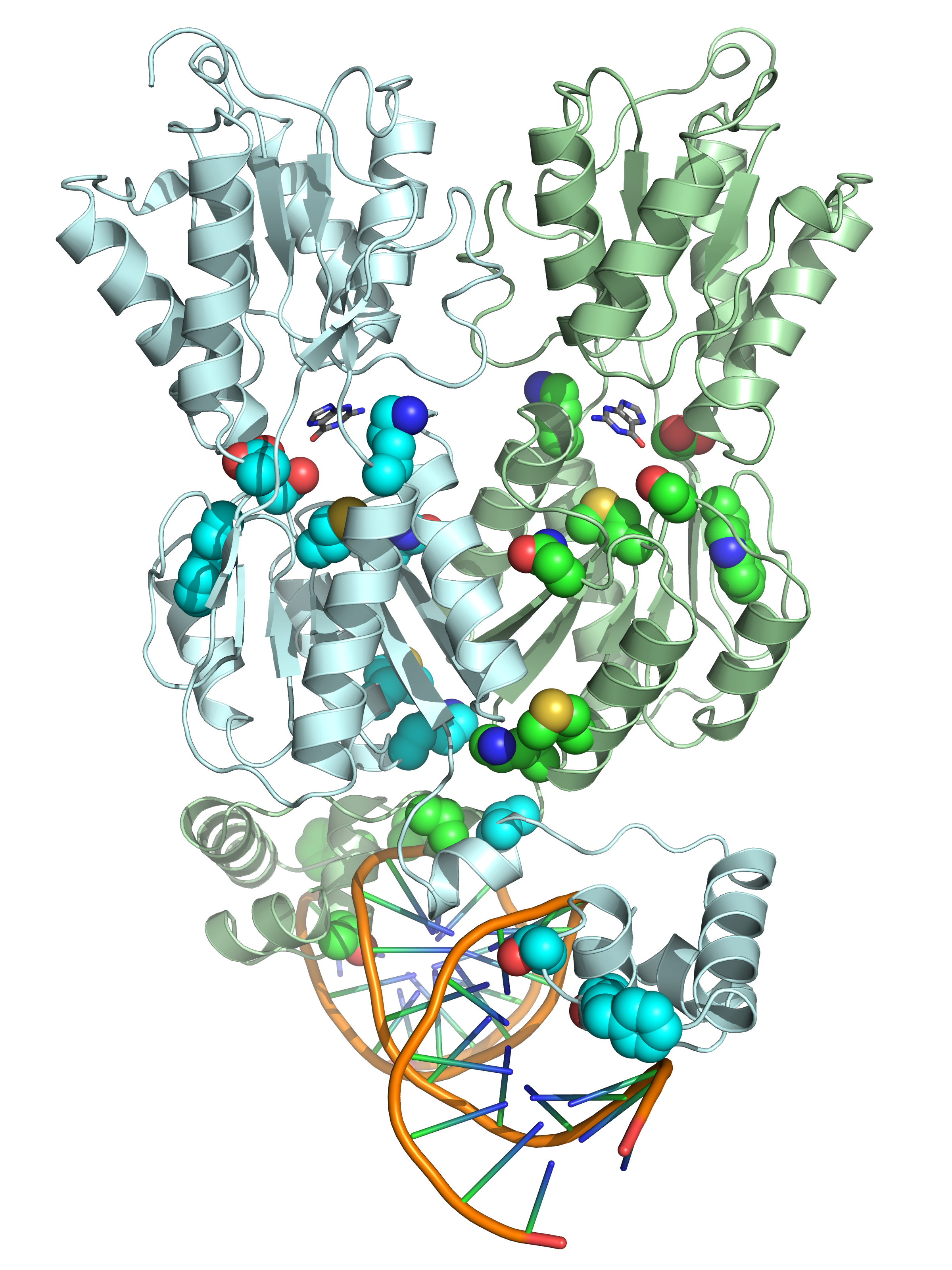

Supplement: S4 Fig — SDPs mapped onto structure 2puc and highlighted in space-filling representation. (PNG) [file pone.0162579.s004.png]

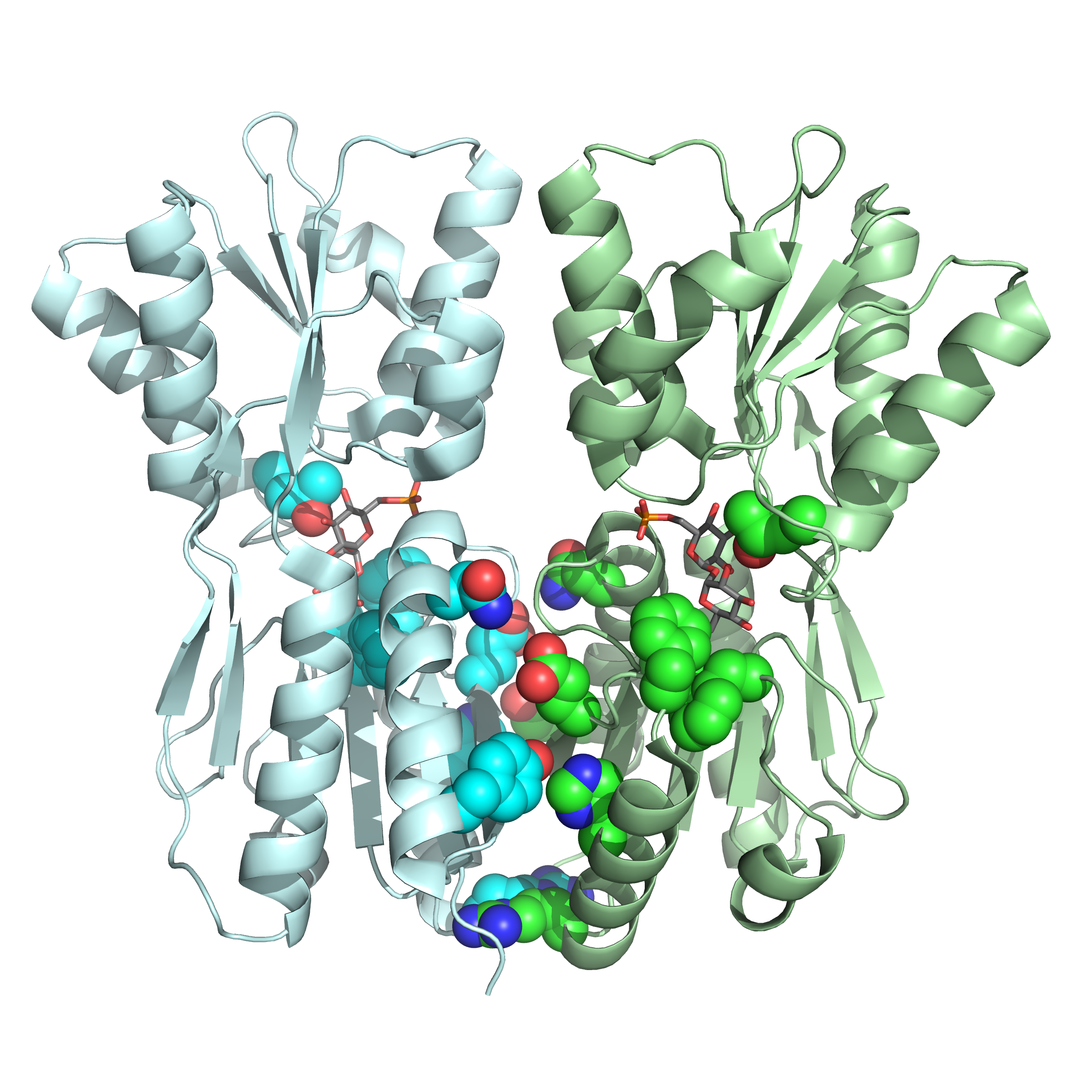

Supplement: S5 Fig — SDPs mapped onto structure 4xxh and highlighted in space-filling representation. Structure only contains N- and C-terminal regulatory subdomains. (PNG) [file pone.0162579.s005.png]
